# Fatigue Performance Evaluation of AZ31B Magnesium Alloy Based on Statistical Analysis of Self-Heating

**DOI:** 10.3390/ma14092251

**Published:** 2021-04-27

**Authors:** Shaofei Guo, Xuesong Liu, Hongxia Zhang, Zhifeng Yan, Hongyuan Fang

**Affiliations:** 1 State Key Laboratory of Advanced Welding and Joining, Harbin Institute of Technology, 92# Xidazhi Street, Nangang District, Harbin 150001, China; s.f.guo@hotmail.com (S.G.); hyfang@hit.edu.cn (H.F.); 2 College of Materials Science and Engineering, Taiyuan University of Technology, 79# Yingze Street, Taiyuan 030024, China; hongxzhang@163.com (H.Z.); yanzhifeng@tyut.edu.cn (Z.Y.)

**Keywords:** self-heating during fatigue, fatigue performance evaluation, magnesium alloy, experiment method

## Abstract

AZ31B magnesium alloy is the experimental material in this study. Considering its anisotropy, fatigue assessment based on self-heating is carried out for both the extrusion direction and the transverse direction. The self-heating behavior in the two orientations is compared. Similar to steels, an obvious inflection point that corresponds to the fatigue limit can be found in the self-heating vs. load curve for AZ31B. A new fatigue limit assessment method is proposed based on a statistical analysis of self-heating data. This method can provide a satisfactory assessment of the fatigue limit for AZ31B in the both orientations.

## 1. Introduction

It is generally believed that the fatigue process includes stages such as the accumulation of damage and the initiation and propagation of cracks [1,2,3,4]. The temperature increase of the material during the fatigue process is called self-heating, which is caused by the conversion of mechanical work applied from the outside into the heat of the material itself [5]. There is a close relationship between self-heating and the evolution of the microstructure within the material related to the accumulation of fatigue damage [6]. At present, many researchers use self-heating to study the fatigue of materials.

In 2010, Amiri and Khonsari [7] proposed a high cycle fatigue life prediction theory by studying the relationship between self-heating and fatigue failure, in which self-heating is represented by the initial rising slope of the temperature evolution curve. The experimental results confirmed that the model proposed by Amiri and Khonsari can provide accurate fatigue life prediction results not only for Stainless Steel 304 but also Aluminum 6061. In 2011, using infrared cameras as a means of temperature measurement, Crupi et al. [8] conducted experiments on three ASTM A 516 gr. 70 steels to investigate the relationship between the temperature evolution curve and the hysteresis loop under low cycle fatigue loading. It was found that the hysteresis loop became stable at the moment when the temperature rise began to enter a long-term stable stage. In the high cycle fatigue life model based on self-heating, the energy parameter (the product of the temperature rise in the stable phase and the fatigue life) related to the fatigue failure capability is usually constant [9,10]. This energy parameter is no longer constant in the improved model for very high cycle fatigue regime because the fatigue life diagram behaves as multistage in very high cycle fatigue regime [11]. The self-heating model with a non-constant energy parameter has been successfully used for the *S*-*N* curve prediction of chromium vanadium alloy cold work tool steel (DIN EN 115CrV3) in a very high cycle fatigue regime [11]. In addition, Wang et al. [12] analyzed the crack growth rate of 316L steel by extracting energy dissipation information from the temperature data of the crack tip area. It demonstrates that the stress-ratio-dependence problem of fatigue crack growth rate can be solved from the perspective of self-heating.

It is particularly worth mentioning that the fatigue limit assessment based on self-heating has the advantages of fast and economical, which is very attractive compared to traditional fatigue test methods [13,14,15]. For example, as one of the classic self-heating methods, the Risitano method [15] theoretically only needs to consume a single specimen to determine the fatigue limit of materials and components. Its step-by-step loading experiment usually only takes a few hours. In contrast, traditional methods such as the staircase method or the *S*-*N* curve method require a lot of repeatability tests. The entire test process takes several weeks and consumes a lot of specimens [16,17]. After proposing the famous Risitano method, the same research team proposed a new fatigue limit assessment method in 2013 [18]. This method is based on the surface temperature evolution of a specimen loaded with a static axial force, which is different from the Risitano method [15] that uses cyclic loading. This new method is suitable for two notched steel specimens. In 2015 [19], a fatigue limit assessment method was proposed and applied to various martensitic stainless steels, which is based on a robust analysis of self-heating data. This method proposed by De Finis et al. [19] can be an automatic fatigue limit assessment method, which is the same as the iteration method of Curà et al. [20]. Meneghetti et al. [21] suggested using the energy dissipated in a unit volume of material as heat as a parameter to characterize fatigue, because this parameter that represents self-heating is considered not to depend on the shape of the specimen and the thermal boundary conditions. A large number of tests were carried out using AISI 304 L stainless steel, involving fatigue limit assessment [22], the effect of average stress [23], and fatigue crack growth [24].

Analyzing self-heating provides a new perspective for fatigue research. At present, these studies are mainly focused on steel. Research on materials other than steel still needs to be enriched. Magnesium alloy has significant anisotropy [25], which is quite different from general steel. Taking this fact into consideration, some questions arise: Is the temperature evolution of magnesium alloy during fatigue affected by material orientation? Can the self-heating theory derived from steel be used to evaluate the fatigue performance of magnesium alloys in different orientations?

The purpose of this paper is to study the fatigue performance of AZ31B magnesium alloy by analyzing the self-heating in both the extrusion direction and the transverse direction. In particular, a data processing method [26] was adopted to ensure that the self-heating of the material can be reflected in a reasonable way. The change of self-heating with time under constant load was investigated by detecting the evolution of temperature rise during high cycle fatigue. The characteristics of self-heating as a function of stress level was discussed by summarizing the experimental results under different loads. On this basis, we analyzed the relationship between self-heating behavior and fatigue performance of AZ31B magnesium alloy. Next, a new fatigue limit assessment method was proposed based on a statistical analysis of self-heating data. At last, the new method was used to evaluate the fatigue limit of AZ31B magnesium alloy.

## 2. Experimental Procedure

The experimental material was AZ31B magnesium alloy in this study. The chemical composition of the present alloy is shown in Table 1 and the basic mechanical properties are shown in Table 2. Meanwhile, the density of AZ31B is 1770 kg/m^3^, the specific heat capacity is 1000 J/(kg·K), and the thermal conductivity is 96 W/(m·K).

**Figure 1:**
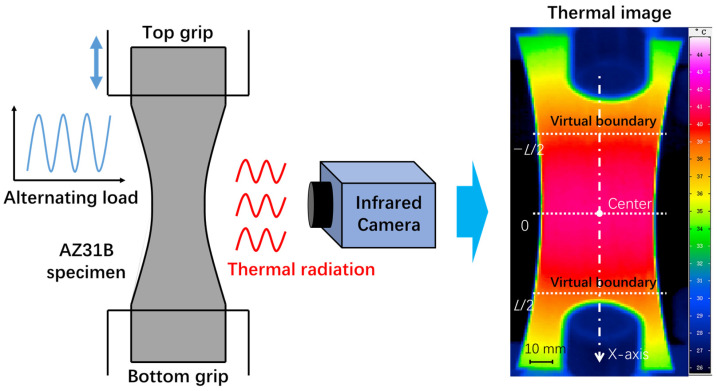
Schematic diagram of the experimental procedure.

Figure 1 is a schematic diagram of the entire experimental procedure. Considering the anisotropy of AZ31B magnesium alloy, we conducted experiments on specimens in both the extrusion direction (ED) and transverse direction (TD). The fatigue test was carried out on a high-frequency resonance fatigue testing machine (PLG-200D, Changchun New Testing Machine Co., Ltd., Changchun, Jilin, China) in accordance with the Chinese national standard GB/T-3075-2008 [27]. Commercial AZ31B magnesium alloy sheet was processed into hourglass-shaped fatigue specimens by spark-cutting. Load levels were classified according to the maximum value of the nominal stress at the center of the specimen with the smallest cross section, *σ*max. The stress control mode was adopted. The load waveform was a sine wave. The load frequency was approximately 100 Hz. The stress ratio was chosen to be 0.1 (*σ*min/*σ*max, where *σ*min is the minimum stress and *σ*max is the maximum stress).

Infrared cameras collect thermal radiation emitted by the object to measure temperature in a non-contact way. In this study, the temperature change of the specimen during the fatigue test was monitored by using an infrared camera (InfraTec VarioCAM hr, InfraTec, Dresden, SN, Germany). A layer of black matt paint was sprayed evenly on the surface of the specimen in order to increase the emissivity and reduce the reflection. Prior to this, the surface of the specimen was smoothed with metallographic sandpaper. The collected temperature data were stored in the form of thermal image at a rate of 50 frames per second. Each pixel in the thermal image represents the temperature value of the corresponding position of the specimen. A large number of thermal images will be generated during the entire fatigue test process. For more experimental details, see reference [26].

The so-called self-heating is the temperature increase inside the material during the fatigue process. On the other hand, the thermal boundary condition describes the heat exchange between the specimen and the external environment. The above two aspects jointly determine the temperature of the specimen collected by the infrared camera [28]. Unfortunately, many factors will cause the thermal boundary conditions to change during the test, such as variable fastening conditions of the specimen [29]. Changes in thermal boundary conditions can interfere with the measurement of self-heating [28]. Therefore, maintaining consistent thermal boundary conditions is a crucial experimental requirement for fatigue research based on self-heating [26].

In this work, a data processing approach [26] was adopted to control the thermal boundary conditions. This is different from the experimental method of adding an external temperature control device in some studies [30]. First, a mathematical model was established based on temperature data measured by the infrared camera, which describes the temperature change in the middle portion of the specimen. Then, based on the superposition principle of the boundary value problem of linear partial differential equations, this thermal diffusion model was split into two parts: temperature rise caused by boundary conditions and temperature rise caused by energy dissipation. On this basis, the temperature rise caused by energy dissipation was further used for fatigue performance evaluation. For a more detailed description of the adopted data processing method, see Ref. [26].

Using the data processing method mentioned above ensures that the temperature on the boundary is always kept at zero [26]. It means that the boundary conditions of the thermal model are fully controlled. In this case, the heat produced by the material itself is the only factor affecting the temperature of the specimen. Therefore, the processed temperature data can be used as a reasonable representative to study the self-heating of AZ31B magnesium alloy under continuous cyclic loading. It should be emphasized that the self-heating behavior analysis and fatigue performance evaluation in the following sections are based on the temperature data after data processing.

## 3. Results and Discussion

### 3.1. Fatigue Performance of AZ31B Magnesium Alloy

**Figure 2:**
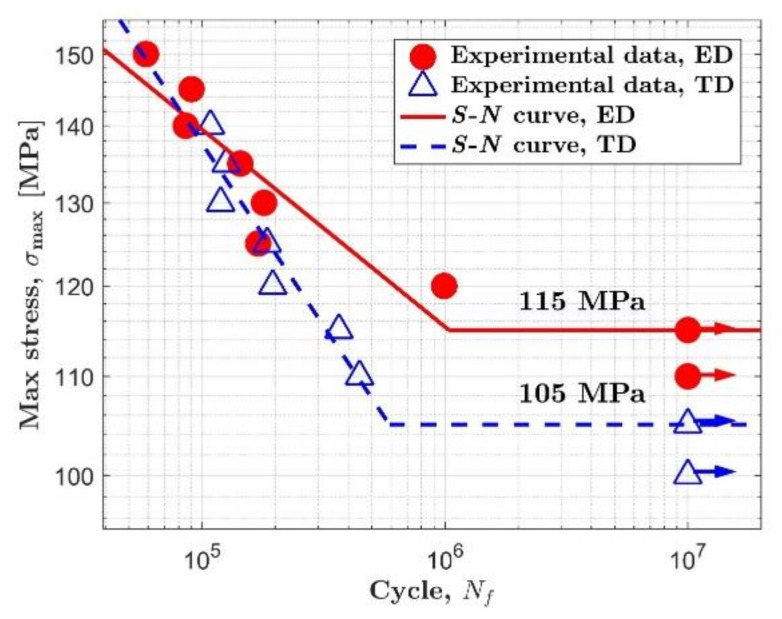
*S*-*N* diagram of AZ31B magnesium alloy.

Figure 2 shows the *S*-*N* diagram of AZ31B magnesium alloy. The data of the extrusion direction (ED) and the transverse direction (TD) are represented by solid circles and open triangles, respectively. Fatigue tests were continued until 10^7^. For the extrusion direction, fatigue failure did not occur when the load level is equal to or less than 115 MPa. Meanwhile, for the transverse direction, fatigue failure did not occur when the load level is equal to or less than 105 MPa.

As can be seen in Figure 2, the knee point can be confirmed in each orientation. *S*-*N* curves of AZ31B magnesium alloy in both orientations show obvious bilinearity, which is similar to the situation with most steels. The AZ31B magnesium alloy used in this study seems to possess a definite fatigue limit regardless of material orientation. Similar *S*-*N* curves were also reported in References [25,31]. So, the fatigue strength under 10^7^ cycles can be defined as the fatigue limit. The fatigue limit of AZ31B magnesium alloy is 115 MPa in the extrusion direction and 105 MPa in the transverse direction.

### 3.2. The Evolution of Temperature Rise under a Single Constant Load

**Figure 3:**
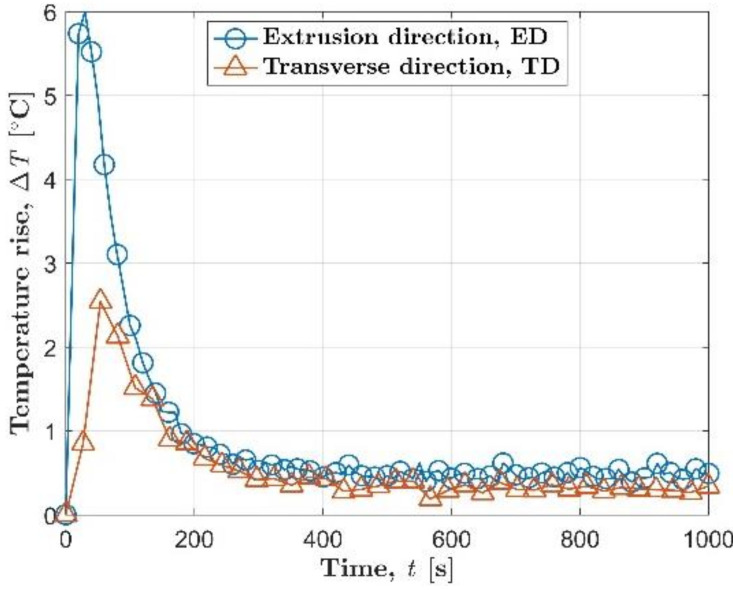
The temperature evolution of AZ31B magnesium alloy under a single constant load of 125 MPa.

The evolution of temperature rise reflects the change of self-heating with time under a single constant load. Figure 3 shows the temperature evolution of AZ31B magnesium alloy under a stress level of 125 MPa. Δ*T* = *T* − *T*_0_ is the temperature rise, where *T* is the mean temperature of the small sampling window at the center of fatigue specimen, and *T*_0_ is the initial temperature of the specimen. For the AZ31B magnesium alloy in the extrusion direction, the temperature increase value Δ*T* rose rapidly to a peak of nearly 6 °C at the very beginning stage. Then, the temperature rise started to drop rapidly from about 30 s. Finally, at about 400 s, the temperature evolution began to enter a relatively stable stage. This stable stage will continue until macro fatigue cracks are initiated [32]. The macroscopic fatigue crack will grow rapidly after initiation. After a short period of crack propagation, fatigue failure will occur, and the fatigue test was stopped.

Under the same stress level of 125 MPa, the temperature evolution in the transverse direction is generally lower than the temperature evolution in the extrusion direction. The maximum temperature increase value of the transverse direction in the entire fatigue test is 2.6 °C, while the maximum temperature increase value of the specimen in the extrusion direction is only 6 °C. During the longer stable stage, the average temperature increase in the transverse direction and the extrusion direction was 0.3 °C and 0.5 °C, respectively. In the initial stage of cyclic loading, the AZ31B in the transverse direction also had a slower temperature rise rate than the extrusion direction, 0.035 °C/s for the transverse direction and 0.28 °C/s for the extrusion direction.

**Figure 4:**
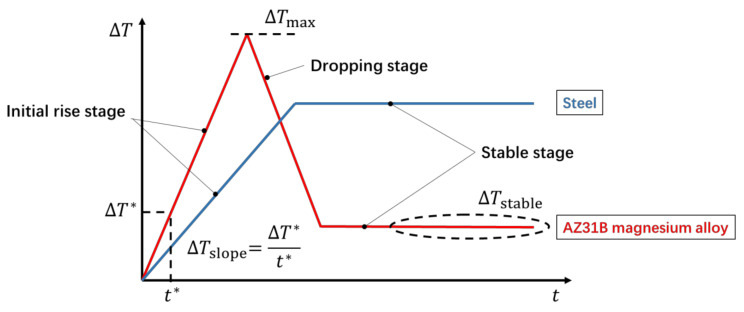
Two different types of temperature evolution curves.

Either in the extrusion direction or in the transverse direction, an obvious hump and the subsequent approximate horizontal line can be observed on the temperature change curve in Figure 3. This is a typical trend shared by AZ31B magnesium alloys under different stress levels. The characteristics of the temperature evolution curve of AZ31B magnesium alloy during fatigue are quite different from those of steel. For engineering steels, the temperature evolution on the specimen surface is characterized by two stages when the applied cyclic load is higher than the fatigue limit: an initial rapid increment, then a plateau region, as shown in Figure 4 [10,15]. However, it is more appropriate to divide the temperature evolution curve of AZ31B magnesium alloy into three stages: the initial temperature rise stage, the temperature drop stage, and the temperature stable stage. Following the work of Doudard et al. [33], the trend of temperature evolution before the appearance of macro fatigue cracks allows one to determine the cyclic hardening type at the micro-scale. The three-stage temperature evolution shows that AZ31B magnesium alloy has undergone cyclic hardening in both orientations [33], which is consistent with the conclusions of the study using mechanical methods [34]. Furthermore, the two-stage temperature evolution curve shows that this type of steel undergoes constant isotropic hardening under cyclic loading [33].

### 3.3. The Relationship between Self-Heating and Load

**Figure 5:**
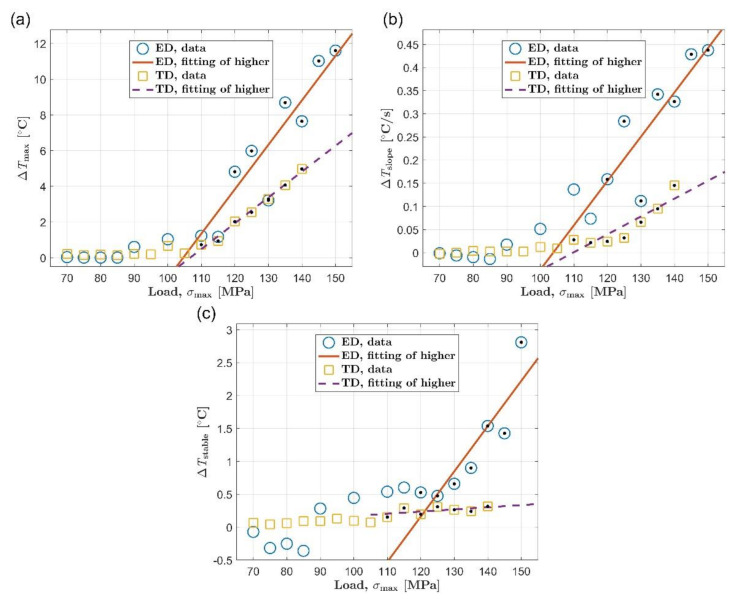
Comparison of self-heating of ED and TD under different loads by using different indicators: (**a**) maximum temperature rise, Δ*T*_max_; (**b**) initial temperature rise slope, Δ*T*_slope_; (**c**) mean temperature rise of stable stage, Δ*T*_stable_.

The relationship between self-heating and load was discussed by summarizing the experimental results under different loads in this section. As illustrated in Figure 4, three commonly used thermal indicators [7,35] are selected to represent the self-heating data under different loads in this study, which are the initial temperature rise slope, Δ*T*_slope_, the maximum temperature increase, Δ*T*_max_, and the temperature increase in the stable stage, Δ*T*_stable_. A dedicated Matlab program was used to automatically calculate these three thermal indicators according to the temperature evolution curve. In particular, the initial temperature rise slope is calculated based on the temperature data of the first 10 s by using a linear fit. The maximum temperature increase takes the maximum value of the entire temperature evolution curve. The stable temperature rise is calculated by averaging the temperature data over a time interval of approximately 100 s during the stable phase. Figure 5 summarizes the self-heating data under different loads, which are respectively represented by the three thermal indicators mentioned above. For the extrusion direction, all three thermal indicators are symbolized by blue circles. For the transverse direction, yellow squares are used to represent the thermal indicators. In addition, all data points above the fatigue limit (determined according to the *S*-*N* diagram in Section 3.1) are marked with black dots for both orientations.

When the load is higher than the fatigue limit, as suggested by Risitano et al. [15], a linear relationship can be used to express the relationship between the self-heating and the load. The result of linear fitting to the data point above the fatigue limit is indicated by the orange solid line and the purple dashed line, respectively, for the extrusion direction and the transverse direction. We compared the linear fitting results of the two orientations. It can be found that obvious anisotropy exists in the relationship between self-heating and load of AZ31B magnesium alloy when the load is above the fatigue limit. Take Figure 5a as an example, in which the maximum value of temperature rise, Δ*T*_max_, is used to represent self-heating behavior. On the one hand, the fitting line in the extrusion direction is above the fitting straight line in the transverse direction. It means that the self-heating of AZ31B in the extrusion direction is higher than that in the transverse direction under the same stress level. On the other hand, the slope of the fitted straight line in the extrusion direction is significantly larger than that in the transverse direction. This shows the fact that growth rate of self-heating with load in the extrusion direction is faster than that in the transverse direction. As shown in Figure 5b,c, the difference between the two orientations can also be observed when using the other two thermal indicators to represent the self-heating of the AZ31B magnesium alloy. This difference is similar to the situation in Figure 5a, so it will not be repeated here.

Unlike the case where the load is higher than the fatigue limit, when AZ31B magnesium alloy bears a load lower than the fatigue limit, the relationship between self-heating and load in the two orientations is not much different from each other. This phenomenon can be observed no matter which thermal indicator is used to represent self-heating data. The self-heating caused by fatigue of AZ31B magnesium alloy seems not significant when the load is below the fatigue limit, and the self-heating hardly increases with the increase of the load.

### 3.4. The Relationship between Self-Heating Behavior and Fatigue Performance

As reported in many literatures [14,15,19], the change in self-heating with load will show an inflection point near the fatigue limit. Correspondence between the inflection point and the fatigue limit has been frequently found in the self-heating behavior of different types of steel [15,19]. It can be seen from Figure 5 that the AZ31B magnesium alloy in the two orientations also conforms to the above regular pattern of steel. For the extrusion direction, the curve of self-heating expressed by the maximum temperature increase exhibits an obvious break at about 115 MPa. When taking the initial temperature rise slope as the thermal indicator, the inflection point of the curve appears between 100 and 115 MPa, and the inflection point of the relationship curve represented by the temperature rise in the stable stage can be identified near 125 MPa. On the other hand, the fatigue limit of the AZ31B magnesium alloy in the extrusion direction is 115 MPa according to the *S*-*N* diagram. The fatigue limit corresponds well to the turning point of the curve of self-heating vs. load for the extrusion direction. For the transverse direction, the inflection points of the self-heating curves represented by the three thermal indicators basically appear around 110 MPa. At the same time, the fatigue limit in this orientation is 105 MPa. Similarly, the fatigue limit and the inflection point are close to each other.

The non-linear relationship between self-heating and load originates from the conversion of self-heating mechanism during fatigue [13,16]. Depending on whether the load is above the fatigue limit, there are two types of self-heating mechanisms: viscoelastic dissipation and microplastic dissipation. When the load is lower than the fatigue limit, viscoelastic dissipation is the main heat generation mechanism. Meanwhile, when the load applied to the material is higher than the fatigue limit, the heat generation mechanism inside the material transforms into microplastic dissipation. Viscoelastic dissipation only produces limited heat per unit time [19]. In contrast, microplastic dissipation will generate a large amount of heat per unit time [16]. The significant difference in heat generation rate between viscoelastic dissipation and microplastic dissipation will result in a sudden and sharp increase in self-heating when the load exceeds the fatigue limit. This will show up as a noticeable break near the fatigue limit.

### 3.5. A New Method for Fatigue Limit Assessment

Viscoelastic dissipation occurs under lower loads, and microplastic dissipation occurs under higher loads. In particular, the fatigue limit is the boundary between these two self-heating mechanisms. This relationship between self-heating behavior and fatigue performance holds not only for various types of steel but also for AZ31B magnesium alloy. For microplastic dissipation, a linear relationship can be used to describe the change in self-heating data with load [15], which can be determined by regression analysis on discrete self-heating data points. The same type of data should obey a consistent statistical law [36]. So, it is reasonable to consider that the self-heating data of microplastic dissipation will be close to the regression line, because the significant difference between the two self-heating mechanisms [19] is likely to cause the self-heating data of viscoelastic dissipation to deviate from the linear law of microplastic dissipation. The self-heating data points belonging to the viscoelastic dissipation should be farther from the regression line than the points of microplastic dissipation. Therefore, we can try to classify the self-heating data based on the distance from the data point to the regression line and then determine the fatigue limit based on the critical point of the two types of self-heating data.

**Figure 6:**
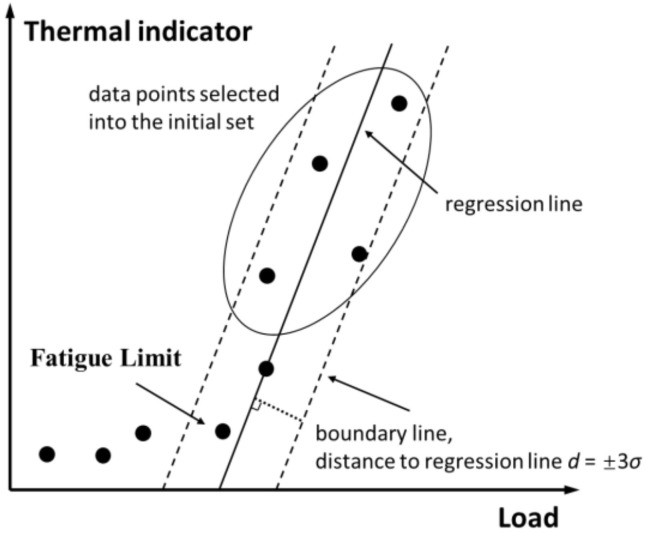
Schematic diagram of the proposed method for fatigue limit assessment.

Follow the above idea, a new fatigue limit evaluation method based on self-heating is developed in this paper. Figure 6 is a schematic diagram of this method.

Step 1 Plot the temperature data obtained under different loads in a rectangular coordinate system. Select some of the self-heating data above the inflection point, as circled with an ellipse in Figure 6. Set the selected data points as the initial set, and perform a linear fit to these data.
Step 2 Perform statistical analysis on the distance from the data points in the initial set to the regression line, and calculate the standard deviation *σ* of these distances. Parallel to the regression line, draw a boundary line on each side of the regression line. The distance between each boundary line and the regression line is three times the standard deviation, 3*σ*.
Step 3 Check the self-heating data points outside the initial set one by one according to the load from high to low. If the current point is within the two boundaries, continue to check the next data point; if the current point is outside the two boundaries, stop the check and consider the load level just before the current point as the fatigue limit.

The regression line together with the two parallel boundary lines constitute an estimation for the statistical law of the microplastic dissipation data points. The regression line comes from a linear fit to the self-heating data points in the initial set, which approximately represents the law of the microplastic dissipation data changing with the load [36]. There are two aspects to pay attention to when determining the initial set. First, the criterion for selecting data points into the initial set is that these points must be clearly above the inflection point of the curve of self-heating vs. load. Only data points that meet such criteria can be identified as microplastic dissipation at the beginning, because microplastic dissipation generates significantly more heat per unit time than viscoelastic dissipation [15]. Second, the number of self-heating data selected into the initial set should be large enough to ensure that the result of linear fitting is a reasonable approximation to the real law [36].

The two parallel boundary lines basically delineate all the areas where microplastic dissipation data may appear. Assume that the distance of all microplastic dissipation obeys a normal distribution with an expected value of zero. Then, the probability that a single microplastic dissipation data point falls within the interval (−3*σ*, +3*σ*) is quite high (over 99%) [36]. If a data point is within this interval, this data point can be judged as microplastic dissipation. On the other hand, in general, the probability of a data point belonging to microplastic dissipation falling outside the interval (−3*σ*, +3*σ*) is very small (less than 1%). Small probability events can be considered impossible [36]. If a data point falls outside the interval (−3*σ*, +3*σ*), this data point can be judged as not belonging to microplastic dissipation but viscoelastic dissipation.

Based on the statistical law represented by the regression line and the two boundary lines, the classification process can be carried out by using a graphical approach in the new method. Check the self-heating data outside the initial set one by one according to the load level from high to low. Stop the test when a data point is found outside the boundary line for the first time. Other data points whose load is lower than the first point outside the boundary line can be considered as viscoelastic dissipation, since the heat generation usually increase monotonically with increasing load [17]. In this way, the self-heating data corresponding to the two self-heating mechanisms can be distinguished. Then, the fatigue limit can be determined as the lowest load level corresponding to the data points of the microplastic dissipation.

This new method is inspired by the work of De Finis et al. [19]. The method of De Finis is to analyze the temperature data below the inflection point to determine the fatigue limit [19]. Different from this, the fatigue limit assessment method introduced in this section is based on the statistical analysis of self-heating data above the inflection point. This part of the self-heating data has a significant temperature rise relative to the initial temperature of the specimen. Random errors will have less influence on the self-heating data selected for analysis in the new method [15].

### 3.6. Fatigue Limit Assessment of AZ31B Magnesium Alloy Using the New Method

The self-heating behavior of AZ31B magnesium alloy has the same characteristics as the self-heating behavior of steel. Therefore, the self-heating method should be equally applicable to the evaluation of fatigue performance of AZ31B magnesium alloy. In this section, the proposed new method is used together with the Risitano method [15] to evaluate the fatigue limit of the AZ31B magnesium alloy in both the extrusion direction and the transverse direction. The Risitano method is one of the classic methods for evaluating the fatigue limit based on self-heating. According to the Risitano method [15], a straight line is used to fit all self-heating data points above the inflection point. Then, find the intersection of the straight line and the abscissa axis at which the thermal indicator is zero. The load corresponding to the intersection point is regarded as the fatigue limit.

**Figure 7:**
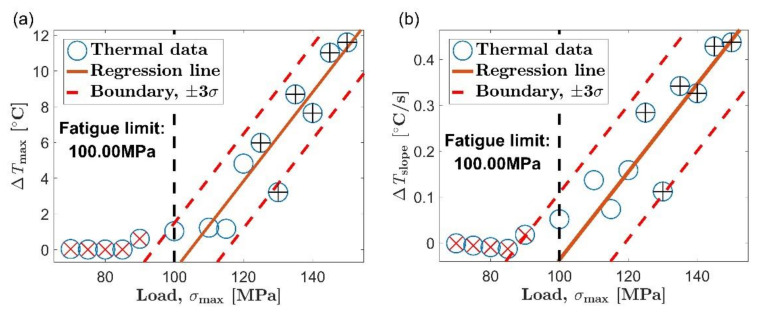

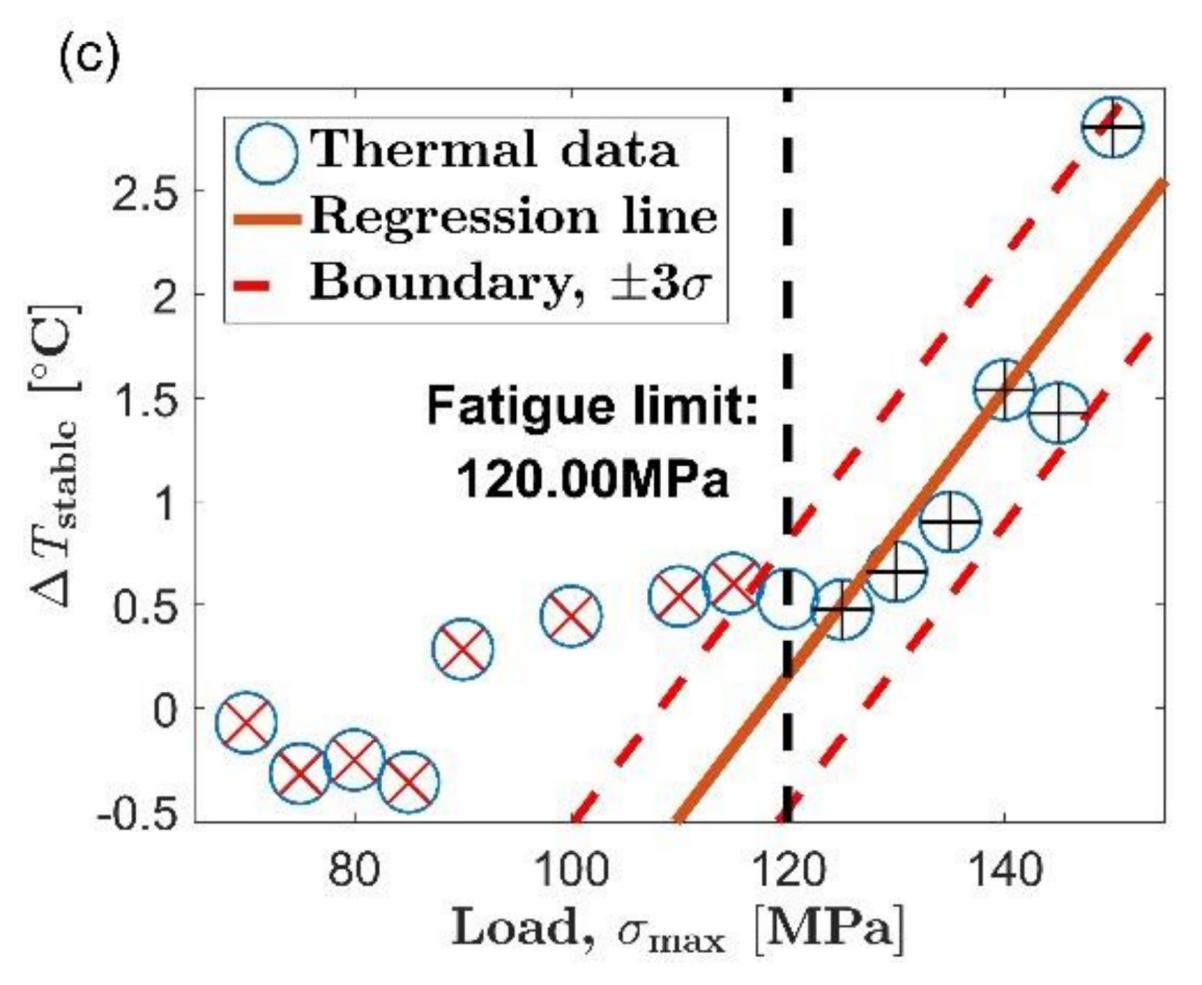
Fatigue limit evaluation using the proposed method, extrusion direction: (**a**) maximum temperature rise, Δ*T*_max_; (**b**) initial temperature rise slope, Δ*T*_slope_; (**c**) mean temperature rise of stable stage, Δ*T*_stable_.

**Figure 8:**
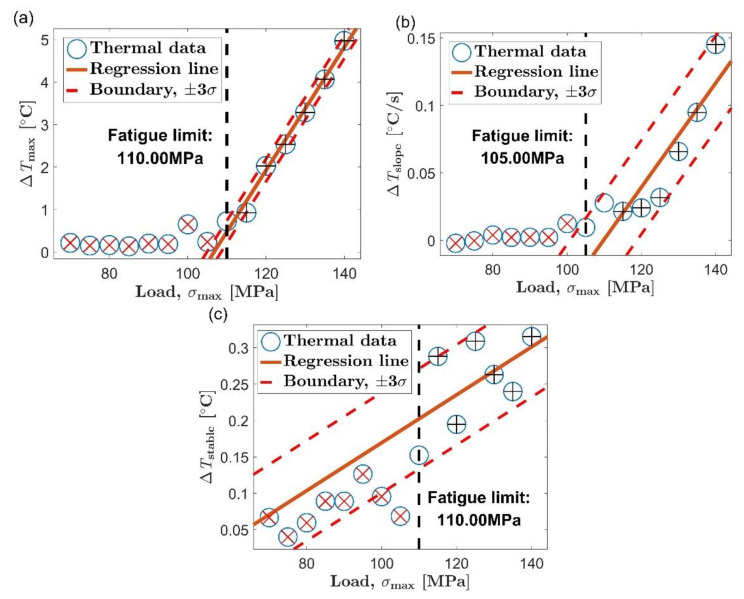
Fatigue limit evaluation using the proposed method, transverse direction: (**a**) maximum temperature rise, Δ*T*_max_; (**b**) initial temperature rise slope, Δ*T*_slope_; (**c**) mean temperature rise of stable stage, Δ*T*_stable_.

Figure 7 and Figure 8 show the evaluation of the fatigue limit in the extrusion direction and transverse direction by the new method, respectively. Six data points are selected into the initial set for linear fitting, which have been marked with black crosses in each picture. The red straight line is the result of a linear fit of the selected data. The two red dashed lines on both sides of the regression line correspond to the boundary lines of plus or minus three standard deviations, respectively. Data points outside the area delineated by the two boundary lines are highlighted with a red diagonal cross, and they are considered not to be microplastic dissipation. The vertical black dashed line indicates the load value corresponding to the fatigue limit.

**Figure 9:**
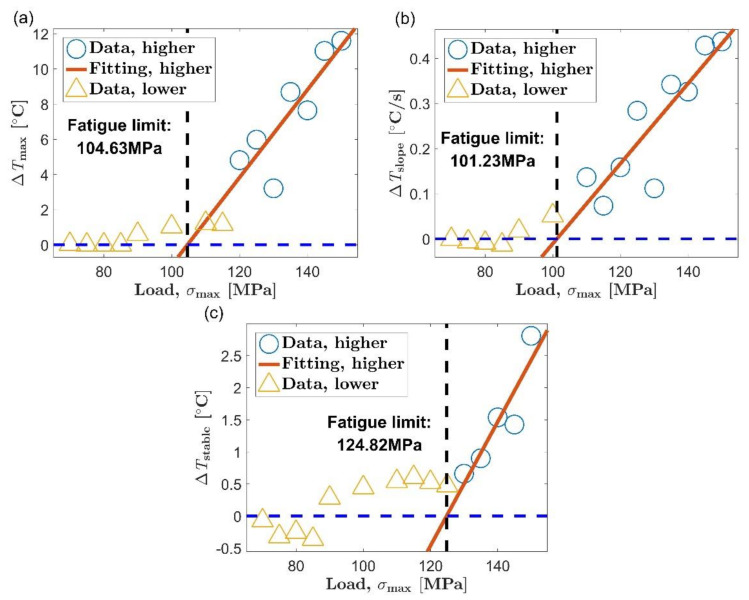
Fatigue limit evaluation using the Risitano method, extrusion direction: (**a**) maximum temperature rise, Δ*T*_max_; (**b**) initial temperature rise slope, Δ*T*_slope_; (**c**) mean temperature rise of stable stage, Δ*T*_stable_.

**Figure 10:**
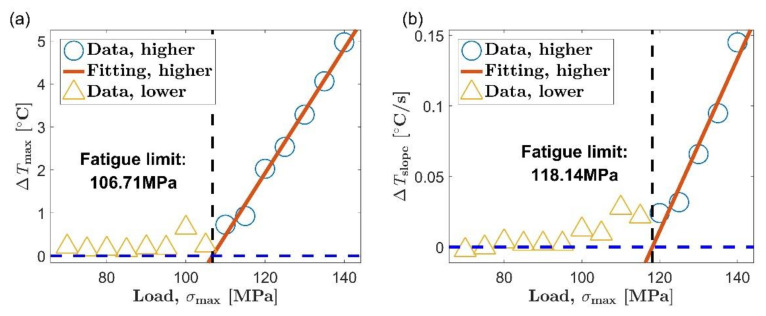

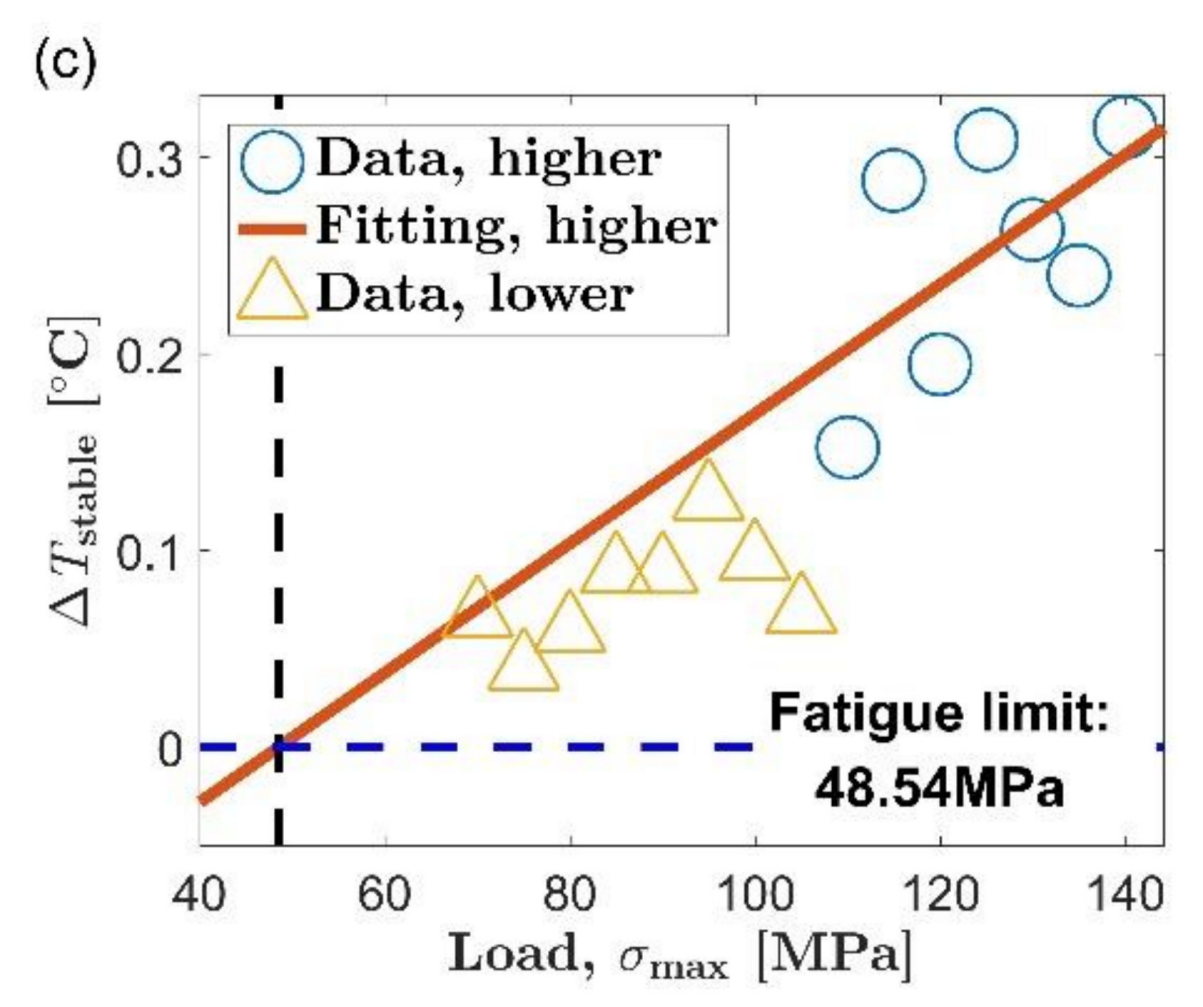
Fatigue limit evaluation using the Risitano method, transverse direction: (**a**) maximum temperature rise, Δ*T*_max_; (**b**) initial temperature rise slope, Δ*T*_slope_; (**c**) mean temperature rise of stable stage, Δ*T*_stable_.

Figure 9 and Figure 10 are the process of evaluating the fatigue limit of AZ31B magnesium alloy using the Risitano method. The position of the inflection point was determined according to the shape of the relationship curve between self-heating and load level. The self-heating data points whose load is higher than the inflection point are represented by blue circles, and the data points below the inflection point are represented by yellow triangles. The red straight line is a linear fit of all the blue circles, and the abscissa axis indicating zero temperature rise is highlighted by a blue dashed line.

The results of the fatigue limit evaluation of AZ31B magnesium alloy based on both methods are summarized in Table 3. The fatigue limit determined according to the traditional *S*-*N* curve is selected as the benchmark value, and the relative error (absolute value) is calculated according to the following formula.

$$error=\left|\frac{\sigma_{\mathrm{SH}}-\sigma_{\mathrm{SN}}}{\sigma_{\mathrm{SN}}}\right|\times 100\%$$

where *σ*_SH_ represents the fatigue limit calculated by self-heating methods and *σ*_SN_ represents the fatigue limit calculated by *S*-*N* curve.

Since the test can be terminated after obtaining the required thermal indicator, the new method has the same advantages as the classic Risitano method; that is, it reduces the time consumed by the experiment. This is the common advantage of the self-heating method over the traditional fatigue testing method [15,17,20]. The average error of the new method for the six test results in Table 3 is 6.66%, and the maximum error is 13.04%. These results demonstrate that the new method can provide a basically satisfactory evaluation for the fatigue limit of AZ31B magnesium alloy, which is true for both the extrusion direction and the transverse direction. At the same time, there is no significant difference between the results of the three selected thermal indicators. It seems that the application of the new method is not limited by the choice of thermal indicators. Moreover, the authors look forward to improving the accuracy of the new method proposed in this paper by adding further tests with subdivided load step size.

The self-heating data adopted by the Risitano method for fatigue limit evaluation are the same as the data used by the new method. However, the Risitano method only gives an accurate evaluation of the first five tests in Table 3. The errors of the first five results of the Risitano method are acceptable. Their average and maximum errors are 8.73% and 12.51%, respectively. Take the third item in Table 3 as an example, in which the stable stage temperature is used as the thermal indicator to evaluate the fatigue limit of the AZ31B magnesium alloy in the extrusion direction. The evaluation process is shown in Figure 9c. Based on the shape of the relationship between self-heating and load, the inflection point of the curve can be determined to be around 125 MPa, which is close to the fatigue limit in the extrusion direction of 115 MPa. Perform a linear fit to the self-heating data above the inflection point and find the intersection of the regression line and the abscissa axis at the zero point. Then, the load corresponding to the intersection point is determined as the fatigue limit. The position of the intersection is 124.82 MPa, which is very close to the position of the inflection point. In this way, an accurate fatigue limit assessment is obtained. The error of the evaluation result relative to the result of the traditional method is 8.54%.

In the sixth test in Table 3, we got a rather poor evaluation result. The evaluation result of the Risitano method is 48.54 MPa, and its error relative to the traditional method is 53.77%. This time, the experimental material is AZ31B magnesium alloy in the transverse direction, and the thermal indicator is still the temperature rise value in the stable stage. Figure 10c illustrates the entire fatigue limit assessment process. The inflection point of the curve of self-heating vs. load can be positioned at 110 MPa; as in the case of Figure 9c, the inflection point is still close to the fatigue limit (105 MPa in the transverse direction). However, different from the case in Figure 9c, the intersection of the regression line and the abscissa axis at the zero point is far away from the inflection point of the curve in Figure 10c.

As already shown in Section 3.3, the fatigue limit of AZ31B magnesium alloy has a good correspondence with the inflection point of its self-heating versus load curve. The fatigue limit can be evaluated as long as the position of the inflection point is determined by a reasonable algorithm. By comparing the evaluation process in Figure 10c with the process in Figure 9c, it can be found that the failure of the fatigue limit assessment in Figure 10c can be attributed to the small slope of the regression line for the self-heating data above the inflection point. The temperature rise during the stable stage was used as the thermal indicator in the both pictures. The same unit allows the slopes of the two fitting lines to be directly compared. The slope of the regression line in Figure 10c is 3.29 × 10 ^−3^, and the slope of the regression line in Figure 9c is 9.65 × 10^−2^. The former is much smaller than the latter. In Figure 10c, it takes a long time for the regression line to intersect the abscissa axis at zero due to its small slope. The consequence is that the intersection point severely deviates from the inflection point of the curve, which results in a large error in the fatigue limit evaluation result. In Figure 9c, because the regression line has a large slope, the fitting line intersects the abscissa axis at zero soon after passing the inflection point of the curve. This leads to an accurate fatigue limit assessment result.

When the load is above the fatigue limit, the slope of the fitting line reflects the change rate of the self-heating data with the load. In most instances, the self-heating changes rapidly with the load, and a relatively steep regression line can be found. However, in some specific cases, the self-heating increases slowly with the load. This will result in a gradual fitting line for the self-heating data above the inflection point. The experimental results show that the Risitano method can definitely be applied to the situation where the self-heating changes rapidly with the load. However, when the slope of the regression line is small to a certain extent, determining the fatigue limit based on the intersection of the fitting lining is likely to cause a failure similar to Figure 10c because the intersection may deviate from the inflection point of the self-heating data curve.

The new method proposed in this paper is to determine the fatigue limit based on the distance between the data point from the to the fitting line, which is an evaluation strategy different from the Risitano method [15]. The two boundary lines are respectively arranged on both sides of the fitting line to the initial set. When the self-heating increases rapidly with the load, the first data point that does not belong to the microplastic dissipation will appear above the boundary line corresponding to positive three times the standard deviation. This is the case in Figure 7a–c and Figure 8a,b. When the self-heating increases slowly with the load, the first data point that does not belong to the microplastic dissipation will appear below the boundary line corresponding to minus three times the standard deviation, similar to the situation in Figure 8c. Using the new method to evaluate the fatigue limit is basically not affected by the change rate of self-heating with load. It allows the new method to be used in the situation mentioned above where the Risitano method is not applicable.

## 4. Conclusions

In this paper, the fatigue performance of AZ31B magnesium alloy is evaluated based on the self-heating caused by cyclic loading. Taking the anisotropy of the AZ31B magnesium alloy into consideration, the analysis of the self-heating is carried out for both the extrusion direction and the transverse direction. Under a single constant cyclic load, the temperature evolution of AZ31B magnesium alloy can be divided into three stages before macroscopic cracks appear: the initial temperature rise stage, the temperature drop stage, and the temperature stabilization stage. This trend of temperature evolution can be observed in the both orientations. However, the temperature evolution in the extrusion direction is higher than that in the transverse direction at all stages. This three-stage temperature evolution curve shows that the AZ31B magnesium alloy has undergone cyclic hardening during the fatigue process.

By summarizing the temperature evolution under different loads, the relationship between self-heating and load was discussed. When the load is higher than the fatigue limit, two differences can be observed between the two orientations of AZ31B magnesium alloy. On the one hand, the extrusion direction has stronger self-heating compared to the transverse direction under the same load level. On the other hand, the increase in self-heating with load in the extrusion direction is faster than that in the transverse direction. When the load is lower than the fatigue limit, the self-heating in the two orientations is relatively limited, and they basically do not change with load.

An obvious inflection point can be found in the self-heating vs. load curve, which corresponds to the fatigue limit of the material. With reference to the research on steel, this correspondence between the inflection point and the fatigue limit of AZ31B magnesium alloy can be attributed to the conversion of heat generation mechanism. Viscoelastic dissipation only produces limited heat per unit time, which is the main heat generation mechanism below the fatigue limit. Meanwhile, microplastic dissipation generates a large amount of heat per unit time, which is the heat generation mechanism above the fatigue limit. The significant difference in heat generation rate between the two self-heating mechanism will result in a sudden increase in self-heating when the load exceeds the fatigue limit.

On the basis of the relationship between self-heating and fatigue performance, a new fatigue limit assessment method was proposed. This method estimates the variation of the self-heating of microplastic dissipation with load through linear fitting. Then, the two self-heating mechanisms are distinguished based on the distance between the data points and the obtained regression line. Finally, the critical point between the two types of self-heating data is identified as the fatigue limit. The new method can provide a basically satisfactory assessment of the fatigue limit of the AZ31B magnesium alloy in the two orientations, and the new method can be applied in a special situation where the classical Risitano method is not applicable. In the future, we look forward to applying this new method to more fields, such as different materials and different temperature ranges.

## Figures and Tables

**Table 1:** Chemical composition of AZ31B magnesium alloy (wt %).

Mg	Al	Zn	Mn	Si	Ca	Cu	Fe	Ni
Bal.	2.8	0.7	0.4	0.1	0.04	0.01	0.005	0.001

**Table 2:** Tensile mechanical properties of AZ31B magnesium alloy.

Material	Tensile Strength	Yield Strength	Elongation
	*σ*m (MPa)	*σ*0.2 (MPa)	A (%)
Extrusion direction, ED	251	145	9
Transverse direction, TD	232	130	12

**Table 3:** Results of fatigue limit obtained by using the proposed method and the Risitano method.

No.	Material	S-*N* Curve	Indicators	Proposed Method		Risitano Method	
		(MPa)		(MPa)	(%)	(MPa)	(%)
1	AZ31B, ED	115	Δ*T*_max_	100	13.04	104.63	9.02
2	AZ31B, ED	115	Δ*T*_slope_	100	13.04	101.23	11.97
3	AZ31B, ED	115	Δ*T*_stable_	120	4.35	124.82	8.54
4	AZ31B, TD	105	Δ*T*_max_	110	4.76	106.71	1.63
5	AZ31B, TD	105	Δ*T*_slope_	105	0	118.14	12.51
6	AZ31B, TD	105	Δ*T*_stable_	110	4.76	48.54	53.77

## Data Availability

No new data were created or analyzed in this study. Data sharing is not applicable to this article.

## References

[1] Karuskevich M.V., Ignatovich S.R., Maslak P., Menou A., Maruschak P., Panin S.V., Berto F. (2016). Multi-purpose fatigue sensor. Part 1. Uniaxial and multiaxial fatigue. Frat. Integrità Strutt..

[2] Karuskevich M.V., Ignatovich S.R., Maslak P., Menou A., Maruschak P., Panin S.V., Berto F. (2016). Multi-purpose fatigue sensor. Part 2. Physical backgrounds for damages accumulation and parameters of their assessment. Frat. Integrità Strutt..

[3] Ivanov Y.F., Alsaraeva K.V., Gromov V.E., Popova N.A., Konovalov S.V. (2015). Fatigue life of silumin treated with a high-intensity pulsed electron beam. Surf. Investig. X-ray Synchrotron Neutron Tech..

[4] Konovalov S., Komissarova I., Ivanov Y., Gromov V., Kosinov D. (2019). Structural and phase changes under electropulse treatment of fatigue-loaded titanium alloy vt1-0. J. Mater. Res. Technol..

[5] Khonsari M.M., Amiri M. (2013). Introduction to Thermodynamics of Mechanical Fatigue.

[6] Connesson N., Maquin F., Pierron F. (2011). Dissipated energy measurements as a marker of microstructural evolution: 316l and dp600. Acta Mater..

[7] Amiri M., Khonsari M.M. (2010). Rapid determination of fatigue failure based on temperature evolution: Fully reversed bending load. Int. J. Fatigue.

[8] Crupi V., Chiofalo G., Guglielmino E. (2011). Infrared investigations for the analysis of low cycle fatigue processes in carbon steels. Proc. Inst. Mech. Eng. Part C J. Mech. Eng. Sci..

[9] Zhang L., Liu X.S., Wu S.H., Ma Z.Q., Fang H.Y. (2013). Rapid determination of fatigue life based on temperature evolution. Int. J. Fatigue.

[10] Fargione G., Geraci A., La Rosa G., Risitano A. (2002). Rapid determination of the fatigue curve by the thermographic method. Int. J. Fatigue.

[11] Crupi V., Epasto G., Guglielmino E., Risitano G. (2015). Thermographic method for very high cycle fatigue design in transportation engineering. Proc. Inst. Mech. Eng. Part C J. Mech. Eng. Sci..

[12] Wang X.G., Ran H.R., Jiang C., Fang Q.H. (2018). An energy dissipation-based fatigue crack growth model. Int. J. Fatigue.

[13] Guo S., Zhou Y., Zhang H., Yan Z., Wang W., Sun K., Li Y. (2015). Thermographic analysis of the fatigue heating process for az31b magnesium alloy. Mater. Des..

[14] Luong M.P. (1998). Fatigue limit evaluation of metals using an infrared thermographic technique. Mech. Mater..

[15] La Rosa G., Risitano A. (2000). Thermographic methodology for rapid determination of the fatigue limit of materials and mechanical components. Int. J. Fatigue.

[16] Guo Q., Guo X., Fan J., Syed R., Wu C. (2015). An energy method for rapid evaluation of high-cycle fatigue parameters based on intrinsic dissipation. Int. J. Fatigue.

[17] Huang J., Pastor M., Garnier C., Gong X. (2017). Rapid evaluation of fatigue limit on thermographic data analysis. Int. J. Fatigue.

[18] Risitano A., Risitano G. (2013). Determining fatigue limits with thermal analysis of static traction tests. Fatigue Fract. Eng. Mater. Struct..

[19] De Finis R., Palumbo D., Ancona F., Galietti U. (2015). Fatigue limit evaluation of various martensitic stainless steels with new robust thermographic data analysis. Int. J. Fatigue.

[20] Cura F., Curti G., Sesana R. (2005). A new iteration method for the thermographic determination of fatigue limit in steels. Int. J. Fatigue.

[21] Meneghetti G. (2007). Analysis of the fatigue strength of a stainless steel based on the energy dissipation. Int. J. Fatigue.

[22] Meneghetti G., Ricotta M. (2012). The use of the specific heat loss to analyse the low- and high-cycle fatigue behaviour of plain and notched specimens made of a stainless steel. Eng. Fract. Mech..

[23] Meneghetti G., Ricotta M., Atzori B. (2016). A two-parameter, heat energy-based approach to analyse the mean stress influence on axial fatigue behaviour of plain steel specimens. Int. J. Fatigue.

[24] Meneghetti G., Ricotta M. (2016). Evaluating the heat energy dissipated in a small volume surrounding the tip of a fatigue crack. Int. J. Fatigue.

[25] Morita S., Ohno N., Tamai F., Kawakami Y. (2010). Fatigue properties of rolled az31b magnesium alloy plate. Trans. Nonferrous Met. Soc. China.

[26] Guo S., Liu X., Zhang H., Yan Z., Zhang Z., Fang H. (2020). Thermographic study of az31b magnesium alloy under cyclic loading: Temperature evolution analysis and fatigue limit estimation. Materials.

[27] ISO (2008). Metallic Materials-Fatigue Testing-Axial-Force-Controlled Method.

[28] Boulanger T., Chrysochoos A., Mabru C., Galtier A. (2004). Calorimetric analysis of dissipative and thermoelastic effects associated with the fatigue behavior of steels. Int. J. Fatigue.

[29] Bouache T., Pron H., Caron D. (2016). Identification of the heat losses at the jaws of a tensile testing machine. Exp. Mech..

[30] Facchinetti M., Florin P., Doudard C., Calloch S. (2015). Identification of self-heating phenomena under cyclic loadings using full-field thermal and kinematic measurements: Application to high-cycle fatigue of seam weld joints. Exp. Mech..

[31] Tokaji K., Kamakura M., Ishiizumi Y., Hasegawa N. (2004). Fatigue behaviour and fracture mechanism of a rolled az31 magnesium alloy. Int. J. Fatigue.

[32] Yan Z.F., Zhang H.X., Wang W.X., He X.L., Liu X.Q. (2014). Temperature evolution in magnesium alloy during static and cyclic loading. Mater. Sci. Technol. Lond..

[33] Doudard C., Calloch S. (2009). Influence of hardening type on self-heating of metallic materials under cyclic loadings at low amplitude. Eur. J. Mech. A Solids.

[34] Yan Z.F., Zhang H.X., Wang W.X., He X.L., Liu X.Q., Wu G.H. (2014). Temperature evolution mechanism of az31b magnesium alloy during high-cycle fatigue process. Appl. Fract. Mech..

[35] De Finis R., Palumbo D., Da Silva M.M., Galietti U. (2018). Is the temperature plateau of a self-heating test a robust parameter to investigate the fatigue limit of steels with thermography?. Fatigue Fract. Eng. Mater. Struct..

[36] Caixing H., Pinmiao T., Jilin W., Shidong L. (2000). Probability Theory and Mathematical Statistics.

